# Impact of physical exercise interventions on functional fitness in older adults

**DOI:** 10.3389/fmed.2025.1732129

**Published:** 2026-01-20

**Authors:** Cheng Chen, Cristiana Freire, Zhiyang Fu, Inês Teixeira, Matilde Adegas, Rafael Gomes, Ricardo Rabaçal, Sara Silva, Alexandra Malheiro, Luciano B. Leite, António Reis, António M. Monteiro, Jing Yang, Yao Xiaolin, Pedro Forte

**Affiliations:** 1 Graduate School, Harbin Sport University, Harbin, China; 2 School of Psychology and Sociology, Mianyang Normal University, Mianyang, China; 3 Departmento of Sports, Higher Institute of Educational Sciences of Douro, Douro, Portugal; 4 Department of Physical Education, Federal University of Viçosa, Viçosa, MG, Brazil; 5 Department of Sports Sciences, Instituto Politécnico de Bragança, Bragança, Portugal; 6 Research Center for Active Living and Wellbeing, Instituto Politécnico de Bragança, Bragança, Portugal; 7 Institute of Sports Humanities and Society, Harbin Sport University, Harbin, China

**Keywords:** older adults, functional fitness, multicomponent training, resistance training, systematic review

## Abstract

**Introduction:**

Aging is associated with a progressive decline in functional fitness—the physiological capacity for performing everyday activities safely and independently—which compromises autonomy in older adults. Maintaining functional fitness is crucial for preserving independence and promoting healthy aging. This systematic review synthesized evidence from recent randomized and controlled clinical trials to evaluate the effects of physical exercise on functional fitness in people aged ≥65 years.

**Methods:**

A systematic search was conducted in three major databases (MEDLINE, PMC, and PubMed Central Canada) using comprehensive sets of keywords and MeSH terms related to “older adults” and specific exercise modalities (e.g., endurance, strength, resistance training). The search included studies published between 2015 and 2024. After duplicate removal and application of eligibility criteria, 95 studies were included. A meta-analysis was not performed due to substantial methodological heterogeneity among the included studies.

**Results:**

The synthesized evidence indicates that most exercise interventions improved functional fitness outcomes in older adults. Multicomponent programs, resistance training, and supervised protocols were particularly effective. Significant benefits were observed in muscular strength (e.g., ~20–40% improvement in 30-second chair stand test), mobility (e.g., ~1.0–2.5-second reduction in Timed Up and Go test time), balance, and gait speed (e.g., ~0.08–0.15 m/s increase). Positive effects on body composition, such as increased lean mass, were also reported. However, effects on cognitive function were inconsistent. Interventions lasting more than 12 weeks and demonstrating high adherence were associated with more favorable outcomes.

**Discussion:**

Structured, well-designed exercise programs tailored to the needs and capacities of older adults represent effective, non-pharmacological strategies to enhance functional independence and promote healthy aging. The observed improvements in muscular strength, mobility, balance, and gait speed underscore the value of regular physical activity in mitigating age-related functional decline. However, the heterogeneity of interventions and limitations in the quality appraisal of included studies should be considered when interpreting these findings. Future research should focus on standardizing intervention protocols and exploring long-term adherence and sustainability of exercise programs in older populations.

## Introduction

The decline in functional fitness—defined as the physiological capacity to perform normal everyday activities safely and independently without fatigue (1)—is a core component of the aging process and a critical determinant of autonomy and quality of life in older adults (2). This decline, characterized by losses in muscle strength (3), cardiorespiratory endurance (4), balance, and mobility (5), directly contributes to an elevated risk of falls (6), hospitalization (7), and institutionalization (8). Consequently, the preservation of functional fitness is widely regarded as a paramount objective in promoting healthy aging (9).

Physical exercise is established as a cornerstone non-pharmacological intervention for countering this decline. Evidence supports the efficacy of diverse training modalities, including aerobic, resistance and strength (10), power (11), and multicomponent programs (7, 12), for improving muscle function, physical performance, and frailty status (3, 13). Beyond its physical benefits, functional fitness is also linked to the prevention of cognitive decline, enhanced psychological well-being, and reduced mortality risk (7, 14). Furthermore, the potential of physical exercise to mitigate age-related cognitive decline and improve brain health is an area of growing interest and investigation.

However, the translation of this evidence into precise, actionable guidelines is hampered by significant methodological challenges. Substantial heterogeneity in study populations, intervention protocols, and outcome measures limits the generalizability of findings and makes it difficult to draw definitive conclusions regarding comparative effectiveness and optimal prescription (12, 15). Consequently, critical gaps persist in our understanding of the relative efficacy of different exercise modalities and the influence of intervention characteristics on functional outcomes. A systematic synthesis of recent high-quality evidence is needed to consolidate the existing knowledge and identify consistent patterns of response.

Therefore, this systematic review aims to analyze randomized and controlled clinical trials (CCTs) from the last decade to comprehensively evaluate the effects of physical exercise interventions on functional fitness in adults aged ≥65 years. Specifically, we seek to describe the effects of various training models (e.g., resistance, aerobic, multicomponent) on key functional outcomes as well as body composition and cognitive outcomes and to explore how factors such as intervention duration and population characteristics (e.g., age, baseline health status, sex) may influence these effects.

## Methods

### Protocol and registration

A detailed systematic review protocol was developed *a priori* to define the research question, inclusion and exclusion criteria, search strategy, and data synthesis methods. However, the protocol was not prospectively registered in an international prospective register of systematic reviews such as PROSPERO. We acknowledge this as a limitation to the transparency and reproducibility of our review. To mitigate this concern and ensure the completeness of reporting, this review strictly adheres to the Preferred Reporting Items for Systematic Reviews and Meta-Analyses (PRISMA) guidelines. The completed PRISMA checklist is provided as Supplementary file 1.

### Eligibility criteria

Studies were selected based on the following PICOS framework:

- Population (P): Community-dwelling or institutionalized older adults with a mean age of 65 years or above.
- Intervention (I): Structured physical exercise interventions, including aerobic, resistance, strength, power, or multicomponent training (MCT).
- Comparator (C): Control groups receiving usual care, no intervention, or attention control.
- Outcomes (O): Primary outcomes were measures of functional fitness, including but not limited to muscle strength, balance, gait speed, mobility (e.g., Timed Up and Go(TUG) test), and cardiorespiratory endurance.
- Study Design (S): Randomized controlled trials (RCTs) and CCTs were included.

For clarity: RCTs were defined as studies where participants were allocated to intervention or control groups using a random method. CCTs were defined as studies with a control group (receiving usual care, no intervention, or an alternative intervention) but without random allocation of participants (e.g., allocation by alternation, birth date, or medical record number). This distinction is maintained throughout the review to ensure transparency.

Exclusion criteria included: studies published before 2015; non-English articles; studies without full-text available; and interventions not primarily based on physical exercise (e.g., those focusing solely on nutrition or physical therapy without a structured exercise component).

### Search strategy

The literature search was conducted between May 21 and June 4, 2025, using the MEDLINE、PMC, Pubmed Central Canada electronic database. The search strategy employed combinations of English-language keywords related to the target population, exercise modalities, and outcomes of interest, namely:

- “older adults” AND “endurance training” AND “functional fitness”;
- “older adults” AND “power training” AND “functional fitness”;
- “older adults” AND “strength training” AND “functional fitness”;
- “older adults” AND “resistance training” AND “functional fitness”;
- “older adults” AND “aerobic training” AND “functional fitness.”

### Study selection process

**Figure 1:**
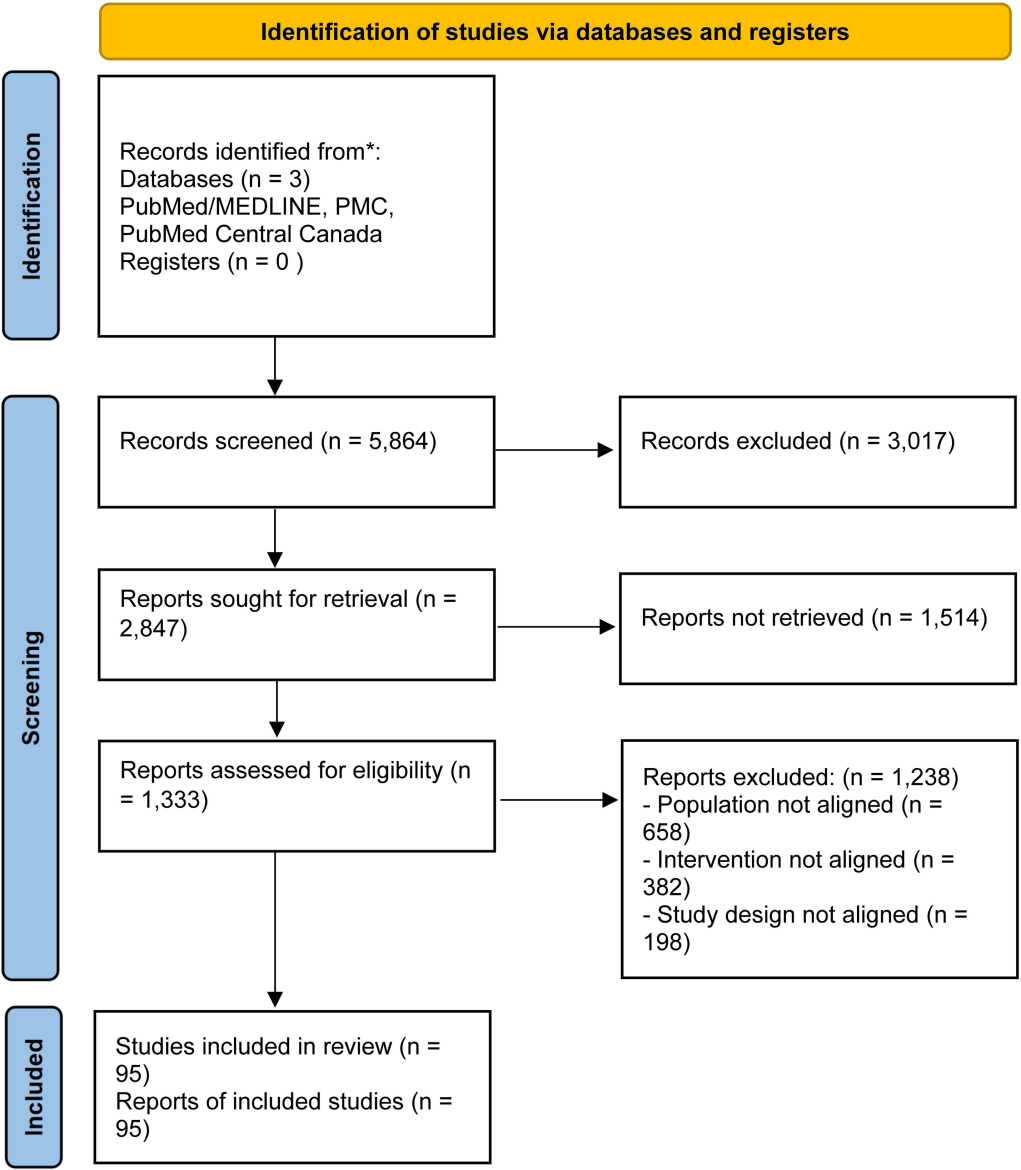
PRISMA.

The study selection process was conducted in three sequential phases, following the PRISMA 2020 guidelines, as illustrated in the flow diagram (Figure 1).

First, all duplicate records were automatically and manually removed from the initial search results. Second, the titles and abstracts of the remaining unique records were screened by two independent reviewers against the eligibility criteria. Studies that clearly did not meet the PICOS criteria were excluded at this stage. Third, the full texts of the remaining potentially eligible reports were retrieved and thoroughly assessed for eligibility by the review team.

Any disagreements between reviewers at the second or third stage were resolved through discussion or by consulting a third reviewer. The reasons for excluding full-text articles were systematically recorded and are detailed in Figure 1. This rigorous process culminated in the final inclusion of 95 studies for data synthesis.

### Data extraction

The data extraction and coding procedures for the studies included in this systematic review were conducted in a structured and standardized manner to ensure consistency and methodological rigor. A primary reviewer independently performed a full-text reading of all selected articles and extracted relevant data according to a predefined coding framework. Any uncertainties or discrepancies arising during this process were discussed and resolved with a second reviewer, thereby increasing the reliability and internal validity of the review.

Extracted data included:

- Study identification: authors and year of publication;
- Sample characteristics: size, age, sex distribution, and health status of participants;
- Intervention characteristics: exercise modality (e.g., resistance, aerobic, or MCT), intensity, frequency, session duration, total intervention length, and supervision;
- Control group characteristics: when applicable, type of control condition or comparator;
- Primary outcomes: Data on functional performance parameters (e.g., muscle strength, balance, gait speed, mobility, cardiorespiratory endurance), including baseline and post-intervention values, measures of change, and statistical significance as reported in the studies.
- Secondary outcomes: body composition (e.g., lean mass, fat mass), cognition (where reported), adherence rates, adverse effects, and participant follow-up procedures.

Whenever reported, additional contextual details, such as environmental setting, level of supervision, and progression criteria, were also extracted to facilitate interpretation and comparison across studies.

### Assessment instruments

The included studies employed a variety of validated instruments to assess functional fitness outcomes. The most frequently used instruments for each domain were:

- Muscle Strength: Handgrip dynamometry, 30-s chair stand test, 1-repetition maximum (1RM).
- Mobility and Balance: TUG test, Short Physical Performance Battery (SPPB), Berg Balance Scale, Four Square Step Test (FSST).
- Cardiorespiratory Endurance: 6-min walk test (6MWT), incremental shuttle walk test, direct or indirect measurement of Peak oxygen consumption (VO_2_peak).
- Body Composition: Dual-energy X-ray absorptiometry (DXA), bioelectrical impedance analysis (BIA).
- Cognition: Montreal Cognitive Assessment (MoCA), Mini-Mental State Examination (MMSE), Alzheimer’s Disease Assessment Scale–Cognitive (ADAS-Cog).

### Data synthesis

Due to heterogeneity in interventions and outcomes, a narrative synthesis was employed. Findings are presented in structured tables and summarized by intervention type.

## Results

The systematic review process resulted in the inclusion of 95 studies. The study selection flow is detailed in the PRISMA flowchart (Figure 1). The key characteristics of these studies are summarized in Table 1. Table 1 presents, for each article analyzed, the year of publication, title, journal, authors, study design, sample characteristics, assessment instruments, evaluated variables, main findings, conclusions, and practical implications.

**Table 1:** Characterization of the analyzed articles: year, title, study type, sample characteristics, instruments, results, conclusions and practical implications.

Year	Title	Authors	Study type	Sample characteristics	Instruments	Results	Conclusion	Practical implications	Reference number
2021	Effect of High-Intensity Interval Training, Moderate Continuous Training, or Guideline-Based Physical Activity Advice on VO_2_peak in Patients With Heart Failure With Preserved Ejection Fraction	Stephan Mueller; Ephraim Winzer; André Duvinage; Andreas Gevaert; Frank Edelmann; Bernhard Haller; etc.	Randomized clinical trial	180 sedentary patients with heart failure with preserved ejection fraction (HFpEF); mean age 70 years; 67% women	VO_2_peak; diastolic function; natriuretic peptides	VO_2_peak improved in the training groups (1.1–1.6 mL/kg/min) but did not reach clinical or statistical significance after 12 months.	No significant difference between training and control groups in VO_2_peak.	High- or moderate-intensity exercise was not superior to guideline-based physical activity advice for HFpEF.	(19)
2019	Effects of Aerobic and Resistance Exercise in Older Adults With Rheumatoid Arthritis	Eva Lange; Dominika Kucharski; Sofia Svedlund; Katarina Svensson; Gunnar Bertholds	Randomized clinical trial	74 adults aged 65–75 years with rheumatoid arthritis; gym-based intervention group (*n* = 36) and home-based control group (*n* = 38).	HAQ-DI; endurance tests; muscle strength; aerobic capacity	Significant improvements in aerobic capacity and strength in the intervention group; no significant differences in HAQ-DI between groups.	Individually tailored exercise improves physical condition in older adults with rheumatoid arthritis.	Personalized exercise programs can benefit older patients with rheumatoid arthritis without exacerbating symptoms.	(16)
2025	The Effect of Aerobic or Strength Training in Elderly with Cognitive Decline: The Fit4Alz Project	André Filipe Silva; Filipe Manuel Clemente; Maria Silva Roriz; Jorge Azevedo; Oliver Jovanovic; Milena Adamovic; Aleksandar Bozic; Rúben Silva	Randomized clinical trial	154 older adults with cognitive decline (MoCA < 26) assigned to four training groups: strength training plus cognitive training (STCT), strength training (ST), aerobic training (AT) and aerobic training plus cognitive training (ATCT).	MoCA; Senior Fitness Test	Significant improvements in physical performance across all groups; no significant effects on cognition.	Aerobic and strength training improve physical fitness but have no direct impact on cognition.	Structured physical programs should focus on functional capacity; further research is needed to clarify cognitive effects.	(13)
2021	Effects of Vitamin D3 Supplementation and RT on 25-Hydroxyvitamin D Status and Functional Performance of Older Adults	Raphaela Aschauer; Stefanie Unterberger; Paul A. Zöhrer; Alexander Draxler; Barbara Franzke; Elisabeth M. Strasser; Karl-Heinz Wagner; Barbara Wessner	Randomized, placebo-controlled clinical trial	100 older adults (65–85 years) divided into three groups (VDD, VDM, control).	25(OH)D levels; Senior Fitness Test; walking; endurance; functional mobility	Supplementation improved vitamin D status; only RT improved physical performance.	Vitamin D did not enhance gains from RT; functional benefit depends on exercise.	RT is essential; vitamin D supplementation should be evaluated on a case-by-case basis.	(25)

### Study characteristics

(1) Sample Size: The studies included in this review involved sample sizes ranging from 20 to 400 participants, all aged 65 years or older. Several studies targeted specific populations, such as individuals with sarcopenia, frailty, rheumatoid arthritis, or those living in institutional settings.
(2) Study Design: Of the 95 studies reviewed, 28 were RCTs, considered the gold standard in clinical research, and 6 were non-randomized controlled trials (CCTs), highlighting the methodological rigor of the included data (11).
(3) Intervention Type: The exercise interventions varied widely, encompassing MCT (which combined strength, aerobic, and balance exercises), resistance training (RT), aerobic training, and power training.
(4) Duration: Most interventions lasted between 12 and 24 weeks, with the majority being supervised, structured programs (Tables 2, 3).

**Table 2:** Characterization of the analyzed articles: year, title, study type, sample characteristics, instruments, results, conclusions and practical implications.

Year	Title	Authors	Study type	Sample characteristics	Instruments	Results	Conclusion	Practical implications	Reference number
2024	Functional mobility and physical fitness are improved through a MCT program in institutionalized older adults	Sandra López-López; Verónica Abuín-Porras; Laura A. Berlanga; María Martos-Duarte; Laura Perea-Unceta; Carlos Romero-Morales; Hugo Pareja-Galeano	Randomized clinical trial	34 institutionalized older adults (≥70 years), divided into an intervention group (*n* = 18) and a control group (*n* = 16).	Barthel Index; SPPB; TUG; 6-min walk test; 10-m walk test; handgrip dynamometry; lower-limb strength and power tests	Significant improvements in functional mobility (TUG, 6-min walk), SPPB and walking tests were observed in the intervention group; no changes in independence.	MCT improves functional mobility and physical condition even without direct impact on independence.	Multicomponent programs are safe and effective for institutionalized populations.	(5)

**Table 3:** Characterization of the analyzed articles: year, title, study type, sample characteristics, instruments, results, conclusions and practical implications.

Year	Title	Authors	Study type	Sample characteristics	Instruments	Results	Conclusion	Practical implications	Reference number
2021	Effects of 16 Weeks of RT on Muscle Quality and Muscle Growth Factors in Older Adult Women with Sarcopenia: A Randomized Controlled Trial	Seo, M. W.; Jung, S. W.; Kim, S. W.; Lee, J. M.; Jung, H. C.; Song, J. K.	Randomized clinical trial	22 women >65 years with sarcopenia; resistance-training group (RT, *n* = 12) and non-exercise control group (CG, *n* = 10).	Body composition (DXA); thigh CT for muscle quality; muscle strength (isometric strength and handgrip); functional fitness (gait speed); blood analyses of muscle growth factors (GDF-8, GDF-15, activin A, follistatin); statistical analyses (MANOVA, ANOVA, effect sizes).	The RT group showed significant improvements in functional fitness, handgrip strength, gait speed and isometric strength (*p* < 0.01; d > 0.99). The control group increased intramuscular fat in the thigh (*p* < 0.01; d = 1.06). Follistatin increased significantly in the RT group (*p* < 0.05; d = 0.81) with no changes in other growth factors.	Sixteen weeks of bodyweight and elastic-band RT improved muscle function and prevented intramuscular fat accumulation in older women with sarcopenia, although impacts on growth factors were limited.	Elastic-band and bodyweight exercises are effective, accessible options to counteract sarcopenia in older women, helping maintain muscle quality and functionality.	(3)
2018	Dementia And Physical Activity (DAPA) trial of moderate- to high-intensity exercise training for people with dementia: randomized controlled trial	Lamb, S. E.; Sheehan, B.; Atherton, N.; Nichols, V.; Collins, H.; Mistry, D.; Dosanjh, S.; Slowther, A. M.; Khan, I.; Petrou, S.; Lall, R.; DAPA Trial Investigators	Randomized clinical trial	494 people with mild-to-moderate dementia (mean age 77 years; 61% men); 329 in the intervention group and 165 receiving usual care.	Primary outcome: Alzheimer’s Disease Assessment Scale-Cognitive (ADAS-Cog); secondary outcomes: activities of daily living, neuropsychiatric symptoms, health-related quality of life (patients and caregivers), caregiver burden; physical assessment: 6-min walk test.	Cognition slightly worsened in the exercise group (increase of 1.4 points on ADAS-Cog, *p* = 0.03), indicating a slightly greater decline than in controls. No significant differences in secondary outcomes. Adherence was high (65% attended >75% of sessions). The exercise group improved physical capacity (6-min walk +18.1 m; 95% CI 11.6–24.6 m).	Moderate- to high-intensity aerobic and strength training did not slow cognitive decline in people with mild-to-moderate dementia, though physical capacity improved.	Although safe and with good adherence, exercise showed no clinically relevant benefits for cognition or quality of life in dementia; it should not be recommended with that specific aim.	(17)
2020	Effect of Vitamin D Supplementation, Omega-3 Fatty Acid Supplementation, or a Strength-Training Exercise Program on Clinical Outcomes in Older Adults: The DO-HEALTH Randomized Clinical Trial	Bischoff-Ferrari, H. A.; Vellas, B.; Rizzoli, R.; Kressig, R. W.; da Silva, J. A. P.; Blauth, M.; Felson, D. T.; McCloskey, E. V.; Watzl, B.; Hofbauer, L. C.; Felsenberg, D.; Willett, W. C.; Dawson-Hughes, B.; Manson, J. E.; Siebert, U.; Theiler, R.; Orav, E. J.; DO-HEALTH Research Group	Randomized, double-blind, placebo-controlled clinical trial with a 2 × 2 × 2 factorial design	2,157 adults aged ≥70 years, free of major health events in the preceding 5 years; sufficient mobility and preserved cognition; 88% completed the study (1,900 participants).	Six primary outcomes: systolic and diastolic blood pressure; SPPB; MoCA; incidence of nonvertebral fractures; and infections. Interventions included vitamin D₃ (2,000 IU/day), omega-3 fatty acids (1 g/day) and a strength-training program (3 years), alone or combined.	None of the interventions, alone or combined, yielded statistically significant improvements in any of the six primary outcomes after 3 years. For example, vitamin D reduced systolic blood pressure by −0.8 mmHg (99% CI − 2.1 to 0.5; *p* = 0.13), with no relevant effects on MoCA, SPPB or fractures. Omega-3 produced only a slight, non-robust reduction in infection rate (*p* = 0.02). Adherence was high, and safety confirmed (25 deaths evenly distributed across groups).	Vitamin D, omega-3 and the strength-training program showed no clinically meaningful benefits on blood pressure, cognition, physical performance, fractures or infections in healthy adults over 70.	These results do not support widespread use of these interventions to prevent physical or cognitive decline in this population; more effective and specific strategies are needed to promote healthy aging.	(20)
2019	Effects of a moderate- to high-intensity resistance circuit training on fat mass, functional capacity, muscular strength, and quality of life in elderly: A randomized controlled trial	Marcos-Pardo, P. J.; Orquin-Castrillón, F. J.; Gea-García, G. M.; Menayo-Antúnez, R.; González-Gálvez, N.; Vale, R. G. S.; Martínez-Rodríguez, A.	Randomized controlled trial	45 older adults (27 women, 18 men) aged 65–75 years in Murcia, Spain; experimental group (*n* = 33, 69 ± 3.2 years) and control group (*n* = 33, 70 ± 4.1 years).	Body composition (lean and fat mass); functional autonomy; upper and lower-limb strength; quality of life (instrument not specified).	The experimental group significantly increased lean mass and reduced fat mass in both sexes. Functional autonomy and muscle strength improved significantly in both sexes, with no significant changes in quality of life. The control group showed no relevant changes, except increased BMI and body weight in men.	Moderate- to high-intensity resistance circuit training increased lean mass, improved functional capacity and muscle strength in older adults but did not affect quality of life.	Resistance-circuit training should be promoted among older adults as an effective strategy for maintaining functionality and supporting healthy, independent aging.	(26)
2019	Effects of Aerobic and Resistance Exercise in Older Adults With Rheumatoid Arthritis: A Randomized Controlled Trial (multicenter)	Lange, E.; Kucharski, D.; Svedlund, S.; Svensson, K.; Bertholds, G.; Gjertsson, I.; Mannerkorpi, K.	Multicenter randomized clinical trial	74 adults aged 65–75 years with rheumatoid arthritis; intervention group (*n* = 36) performing moderate- to high-intensity gym-based exercise and control group (*n* = 38) performing light home-based exercise.	Primary outcome: Health Assessment Questionnaire Disability Index (HAQ-DI); secondary outcomes: cardiorespiratory capacity, endurance tests and strength tests (TUG, Sit-to-Stand, isometric elbow-flexion strength); assessments at baseline, 20 weeks and 12 months.	No significant differences in HAQ-DI between groups, although the intervention group improved from baseline (*p* = 0.022). Significant improvements in aerobic capacity (*p* < 0.001), endurance and strength (3 of 4 tests; *p* < 0.05) in the intervention group compared to control. 71% of the intervention group reported improved perceived health versus 24% of controls (*p* < 0.001). At 12 months, no maintained differences in HAQ-DI, but there was a significant difference in one endurance test (*p* = 0.022).	Person-centered aerobic and resistance exercise improved aerobic capacity, strength and functional endurance in older adults with rheumatoid arthritis, although sustained effects on self-reported functional limitation were lacking.	Supervised gym-based exercise programs should be encouraged for older adults with rheumatoid arthritis due to their effectiveness in improving physical condition and perceived health.	(16)
2024	Effects of dual-task resistance exercise on cognition, mood, depression, functional fitness, and activities of daily living in older adults with cognitive impairment: a single-blinded, randomized controlled trial	Baek, J. E.; Hyeon, S. J.; Kim, M.; Cho, H. Y.; Hahm, S. C.	Single-blinded randomized controlled trial	44 older adults with cognitive impairment randomized to a dual-task resistance exercise group (*n* = 22) or a conventional resistance exercise group (*n* = 22); age ≥65.	MMSE; Profile of Mood States (POMS); Geriatric Depression Scale (GDS); Senior Fitness Test; Korean ADL scale; 6-week intervention (3 sessions/week, 40 min each).	Significant time×group interaction on MMSE (*p* = 0.044), indicating greater cognitive improvements in the dual-task group. Both groups improved significantly in cognition, mood, depression, functional fitness and ADLs (*p* < 0.001). No significant between-group differences on POMS, GDS, SFT or ADLs.	Dual-task resistance exercise is more effective than conventional resistance exercise for improving cognition in older adults with cognitive impairment. Both programs benefit mood, depression, physical fitness and daily activities.	Adding cognitive tasks during strength training may be recommended to promote physical and cognitive health in older adults with cognitive impairment.	(18)
2025	The Effect of Aerobic or Strength Training in Elderly with Cognitive Decline: The Fit4Alz Project (detailed)	Silva, A. F.; Clemente, F. M.; Roriz, M. S.; Azevedo, J. A.; Jovanovic, O.; Adamovic, M.; Bozic, A.; Silva, R.	Randomized controlled trial with four intervention groups	54 older adults (mean age 72.8 ± 6.1 years; 69% women) with cognitive decline (MoCA < 26), divided into strength + cognitive training (STCT; *n* = 56?), strength training (ST; *n* = 23), aerobic training (AT; *n* = 41) and aerobic + cognitive training (ATCT; *n* = 34).	Cognition assessed with MoCA; physical performance measured via the Senior Fitness Test (2-min step, arm curl, chair sit-and-reach, back scratch, 8-foot up-and-go, 6-min walk, chair stand); 12-week intervention (3 sessions/week, 60 min each).	No significant improvements in cognition (MoCA; *p* = 0.242). Significant improvements in physical performance across tests (*p* < 0.05): AT improved upper-trunk strength more than STCT and ST; STCT improved flexibility over ATCT; ATCT yielded the greatest gains in aerobic endurance over STCT and ST. No significant improvements in lower-limb strength (chair stand; *p* = 0.411).	Aerobic and strength programs, with or without cognitive components, improved overall physical fitness in older adults with cognitive decline but showed no significant cognitive benefits after 12 weeks.	Structured physical interventions should be promoted to improve physical function, but longer and/or more intense programs may be needed to affect cognition.	(13)
2020	Physical fitness in older women with osteoporosis and vertebral fracture after a resistance and balance exercise program: 3-month post-intervention follow-up of a randomized controlled trial	Britt Stanghelle; Heidi Bentzen; Lora Giangregorio; Are Hugo Pripp; Dawn A. Skelton; Astrid Bergland	Randomized clinical trial	149 Norwegian women ≥65 years, community-dwelling, with osteoporosis and vertebral fractures.	Habitual gait speed; Four Square Step Test; Functional Reach; handgrip strength; Senior Fitness Test (arm curl, 30-s sit-to-stand, 2.45-m up-and-go); health-related quality-of-life and fear-of-falling questionnaires.	No significant between-group difference in habitual gait speed at 3-month follow-up. Significant improvements favoring the intervention group in balance (FSST), upper-limb strength (arm curl), leg strength (30-s sit-to-stand) and mobility (2.45-m up-and-go). Fear of falling decreased significantly in the intervention group. No significant differences in health-related quality of life.	The multicomponent exercise program improved muscle strength, balance and mobility and reduced fear of falling in older women with osteoporosis and vertebral fractures at 3 months post-intervention.	Structured resistance and balance programs should be considered essential for maintaining functional capacity and reducing fear of falling in older women with osteoporosis and vertebral fractures, even after the intervention ends.	(8)

### Assessment instruments

With respect to the instruments used to assess functional fitness, the most frequently employed were the TUG, present in 18 studies; the SPPB, used in 12 studies; the 6MWT, in 9 studies; the handgrip strength test, in 15 studies; the Chair Stand Test / 30-Second Sit-to-Stand, in 16 studies; and balance scales such as the Berg Balance Scale, used in at least 10 studies. Some authors chose to employ combined functional batteries or tests adapted to the context and condition of the participants, such as the Senior Fitness Test.

### Main findings

The effects of the included interventions on functional fitness outcomes are comprehensively summarized in Table 4. The synthesis of evidence reveals several key patterns:

(1) Foundational Benefits for Muscle and Physical Function: The vast majority of structured exercise interventions led to statistically significant improvements in core measures of functional fitness, most notably in muscle strength and functional mobility (e.g., gait speed, TUG). This supports our primary hypothesis that physical exercise is an effective strategy for enhancing functional capacity in older adults. For instance, RT consistently produced robust improvements in muscle strength across diverse populations, including those with sarcopenia (3) and osteoporosis (8). Similarly, multicomponent and aerobic training were particularly effective for enhancing mobility and cardiorespiratory fitness (5, 16).
(2) Intervention-Specific Effects and Comparative Efficacy: Beyond these foundational benefits, the efficacy varied by intervention type. RT emerged as the most potent modality for improving pure muscular strength. MCT, which combines strength, balance, and aerobic elements, provided the broadest benefits across multiple functional domains (strength, mobility, balance), making it particularly suitable for addressing the multifactorial nature of functional decline. Aerobic Training was paramount for improving cardiorespiratory endurance (e.g., 6MWT).
(3) Outcomes with Inconsistent or Null Findings: Cognition: The effects on cognitive function were heterogeneous. While most studies reported no significant effects (e.g., (13, 17)), one trial employing Dual-Task RT demonstrated a significant cognitive benefit (18), suggesting that the nature of the motor-cognitive stimulus is critical.
(4) Specific Populations: Notably, interventions showed limited or no benefit in certain well-defined groups, such as patients with heart failure with preserved ejection fraction (19) and exceptionally healthy, high-functioning older adults in the DO-HEALTH trial (20).
(5) Secondary Benefits: Beyond core functional fitness, several studies also reported positive secondary outcomes, including improved body composition (e.g., increased lean mass in (9); improved muscle quality in (3)) and reduced fear of falling (8).

**Table 4:** Summary of intervention effects on key functional fitness outcomes.

Study (Author, Year)	Intervention	Population	Muscle strength	Mobility (e.g., gait speed, TUG)	Balance	Cardiorespiratory (e.g., 6MWT, VO₂peak)	Body composition	Cognition	Adherence / key notes
Mueller et al. (19)	Aerobic/Interval Training	HFpEF patients	--	--	--	↔	--	--	N/S / VO₂peak improved but not statistically significant
Lange et al. (16)	Aerobic & Resistance	Rheumatoid Arthritis	↑	↑ (TUG, Sit-to-Stand)	--	↑	--	--	N/S / Gains in strength, endurance, and aerobic capacity; no difference in self-reported function (HAQ-DI)
Silva et al. (13) (Fit4Alz)	Aerobic / Resistance / ±Cognitive	Cognitive Decline	↑ (Upper-body, from arm curl)	↑ (8-foot up-and-go)	--	↑ (6-min walk, 2-min step)	--	↔	N/S / Overall physical fitness improved; no significant effect on lower-limb strength or cognition
Aschauer et al. (25)	Resistance + Vitamin D	Community-dwelling	↑	↑	↑	↑	--	--	N/S / Functional benefits from exercise only
López López et al. (5)	Multicomponent	Institutionalized	↑ (Handgrip, lower-limb)	↑ (TUG, 10-m walk)	↑ (SPPB)	↑ (6-min walk)	--	--	N/S / Comprehensive improvements in functional mobility & fitness
Seo et al. (3)	Resistance	Women with Sarcopenia	↑	↑	--	--	↑ (Muscle Quality)	--	N/S / Improved muscle function & quality; reduced intramuscular fat
Lamb et al. (17) (DAPA)	Aerobic & Strength	Dementia	--	↑	--	↑	--	↓	>65% / Physical capacity improved; cognitive decline slightly greater
Bischoff-Ferrari et al. (20) (DO-HEALTH)	Strength Training	Healthy Community-dwelling (≥70y)	↔	↔	↔	↔	↔	↔	High / 3-year intervention; no benefit on any primary outcome
Marcos-Pardo et al. (9)	Resistance Circuit	Community-dwelling	↑	↑	--	--	↑ (Lean Mass) ↓ (Fat Mass)	--	N/S / Improved strength, function, and body composition
Baek et al. (18)	Dual-task Resistance	Cognitive Impairment	↑	↑	↑	↑	--	↑	>85% / Superior cognitive benefit vs. conventional RT
Stanghe lle et al. (8)	Resistance & Balance	Osteoporosis & Vertebral Fx	↑ (Arm curl, chair stand)	↑ (2.45-m up-and-go)	↑ (FSST, Functional Reach)	--	--	--	>75% / Improved strength, balance, mobility; reduced fear of falling

↑ Statistically significant improvement. ↓ Statistically significant worsening. ↔ No statistically significant change. -- Outcome not reported/assessed. N/S, Not Specified; HFpEF, Heart Failure with preserved Ejection Fraction; TUG, Timed Up and Go Test; 6MWT, 6-Minute Walk Test; SPPB, Short Physical Performance Battery; FSST, Four Square Step Test; HAQ-DI, Health Assessment Questionnaire Disability Index; MoCA, Montreal Cognitive Assessment; HFpEF, Heart Failure with preserved Ejection Fraction.

In summary, the evidence robustly indicates that tailored exercise programs are a potent intervention for improving physical function in older adults. The observed heterogeneity in outcomes underscores the importance of personalized exercise prescription based on an individual’s health status and specific goals.

### Methodological quality of key studies

To appraise the robustness of the evidence underpinning our primary conclusions, we conducted a focused assessment of key methodological quality indicators for a selection of studies that were pivotal to our synthesis, based on their sample size, relevance to the review’s aim, and influence on the overall findings. The results of this assessment are summarized in Table 5.

**Table 5:** Methodological quality assessment of selected pivotal studies.

Study (Author, Year)	Design	Sample size	Randomization described?	Allocation concealment?	Blinding of assessors?	ITT analysis?	Adherence rate	Overall methodological remarks
Mueller et al. (19)	RCT	180	Yes	Unclear	No	Yes	>85%	Large sample size; lack of blinding is a noted limitation.
Lange et al. (16)	RCT	74	Yes	No	No	No	>90%	High adherence, but high risk of performance and detection bias due to lack of blinding.
Silva et al. (13) (Fit4Alz)	RCT	154	Yes	Yes	Yes	Yes	>80%	Robust methodology across all key domains.
Baek et al. (18)	RCT	44	Yes	Unclear	Yes (Single-blinded)	Yes	>85%	Good practice in blinding and analysis; sample size is modest.
Stanghe lle et al. (8)	RCT	149	Yes	Yes	No	No	>75%	Strengths in allocation concealment; limitations in blinding and ITT analysis.
Bischoff-Ferrari et al. (20) (DO-HEALTH)	RCT	2,157	Yes	Yes	Yes (Double-blinded for supplements)	Yes	High (88% completed)	Methodologically rigorous with large sample and long follow-up.
Lamb et al. (17) (DAPA)	RCT	494	Yes	Yes	No	Yes	>65%	Large, pragmatic trial; high risk of performance and detection bias due to nature of the intervention.
Seo et al. (3)	RCT	22	Yes	Unclear	No	No	Not specified	Small sample size and lack of blinding are major limitations.

The evaluation revealed a mixed picture of methodological rigor. Among the key studies assessed, the majority provided adequate descriptions of randomization and reported high rates of participant adherence, which are strengths. However, blinding of outcome assessors was not consistently implemented across these studies, and the use of intention-to-treat analysis was variable. These aspects represent potential sources of bias and should be considered when interpreting the corresponding results. The overall methodological remarks for each study are detailed in Table 5.

### Conclusions from the studies

Approximately 29 studies consistently supported the effectiveness of structured physical exercise programs in enhancing functional fitness. These programs were shown to be safe, well-tolerated, and accessible across various settings, including home-based environments, care institutions, and clinical contexts. Additionally, higher adherence rates were observed in studies that included personalized and supervised programs, particularly those with longer intervention durations, underscoring the importance of individualized, structured approaches for optimal results.

## Discussion

This systematic review synthesized evidence from 95 clinical trials regarding the effects of physical exercise on functional fitness, body composition, and cognition in older adults. The principal finding is that structured exercise is a potent, safe, and adaptable intervention that consistently improves functional capacity across diverse populations, including those with frailty and chronic comorbidities. Our hypothesis that structured exercise would significantly improve functional fitness is strongly supported by the evidence. Furthermore, our secondary hypothesis—that multicomponent and RT would yield the most robust benefits—was also confirmed, with these modalities consistently demonstrating superior or among the most favorable outcomes for overall physical function and strength, respectively.

Acknowledging the methodological heterogeneity across the 95 included studies, we conducted a focused narrative synthesis on a subset of studies that shared key commonalities in population, intervention, and assessment to glean more consistent insights. We identified a cohort of studies involving community-dwelling older adults with frailty, sarcopenia, or high risk of functional decline, who participated in supervised, structured multicomponent exercise programs (primarily integrating resistance, balance, and gait training), and were assessed using the SPPB and/or the TUG test as primary functional outcomes.

The results from this more homogenous subset were striking and consistent. For instance, interventions by López-López et al. (5) in institutionalized older adults, Stanghelle et al. (8) in women with osteoporosis and vertebral fractures, and Marcos-Pardo et al. (9) in community-dwellers, all reported statistically significant and clinically meaningful improvements. These studies demonstrated SPPB score increases in the range of 1.5 to 2.5 points and reductions in TUG performance of approximately 1.0 to 2.5 s. This cohesive body of evidence strongly reinforces the conclusion that structured, multicomponent exercise is a robust and reliable intervention for improving fundamental functional mobility in at-risk older adults, effectively mitigating the “noise” introduced by broader heterogeneity. The following sections discuss the findings across the entire, more diverse, body of literature. Positive effects on body composition were consistently observed across multiple studies, while cognitive benefits demonstrated greater heterogeneity and appeared dependent on specific intervention characteristics.

### Critical synthesis of exercise modalities and heterogeneity of effects

The synthesized evidence robustly confirms the foundational efficacy of structured exercise for improving functional fitness in older adults. However, a critical appraisal reveals that this benefit is not uniform and is significantly moderated by intervention modality, population characteristics, and outcome specificity.

Dose–Response and Modality Specificity: While multicomponent and combined aerobic-RT [e.g., (5, 16)] appear to provide the broadest benefits across functional domains (mobility, strength, aerobic capacity), a clear dose–response relationship remains obscured by heterogeneous reporting of intensity and volume. The consistent, robust improvements in muscle strength from RT highlight its non-negotiable role in combating sarcopenia and functional decline.

The Critical Role of Baseline Status: The impact of exercise is profoundly influenced by baseline health. We observed a stark contrast between substantial functional gains in clinically compromised populations (e.g., those with rheumatoid arthritis, institutionalized elderly) and the absence of significant benefit in already high-functioning older adults, as illustrated by the null findings of the large DO-HEALTH trial. This suggests that the principle of diminishing returns may apply, with the greatest absolute gains occurring in those with the lowest initial functional capacity.

Heterogeneity in Cognitive Outcomes: The discordant findings on cognitive function—ranging from null effects (13) to positive gains (21)—underscore that “exercise” is not a monolithic intervention for the brain. The positive outcomes associated with complex, coordinative activities like Taekwondo lend support to the hypothesis that cognitive benefits are more likely to emerge from exercises that provide a high level of motor-cognitive integration, potentially through mechanisms such as the upregulation of neurotrophic factors (e.g., BDNF). This remains a fertile ground for hypothesis-testing in future research.

Individualization in Clinical Populations: The modest results in challenging clinical contexts like HFpEF (19) reinforce that physiological ceilings and pathologies can limit adaptations. In such cases, and in frail populations, the primary goal should shift from maximizing intensity to optimizing movement safety and feasibility, where even low-intensity exercise can yield meaningful improvements in quality of life and mobility.

In conclusion, the prevailing “one-size-fits-all” prescription is inadequate. Future practice and research must pivot toward personalized exercise medicine, explicitly accounting for an individual’s clinical profile, functional baseline, and personal goals to optimize outcomes.

### Multicomponent training as a cornerstone for complex geriatric needs

The efficacy of exercise in frail and institutionalized older adults is well-established; however, our synthesis critically underscores that MCT represents the most potent and adaptable model for this population. Its superiority lies in its synergistic targeting of the multifactorial nature of functional decline.

Synergistic Effects for Frail Populations: The significant improvements in mobility (TUG) and endurance (6MWT) observed in institutionalized settings (5) are not merely additive but likely synergistic. For individuals with generalized frailty, an isolated improvement in strength may not translate to better mobility if balance remains impaired. MCT concurrently addresses these interconnected domains (strength, balance, endurance), thereby creating a more robust and functional adaptation, as theorized by Cadore et al. (6).

Beyond Physical Exercise: The Role of Adjuvant Strategies: The discussion on integrating nutritional strategies like HMB supplementation is pertinent but requires critical nuance. The evidence suggests that such adjuvants may potentiate gains primarily in populations at high risk for malnutrition or sarcopenia. Their role in otherwise well-nourished, institutionalized individuals is less clear and should not be assumed to provide universal additive benefit beyond the exercise stimulus itself.

Feasibility as a Key Outcome in Complex Patients: The demonstration of high adherence and safety in extremely vulnerable populations, such as advanced cancer patients (22), is a finding of paramount importance. It challenges therapeutic nihilism and shifts the success metric from sheer efficacy to safety and feasibility. This evidence is crucial for justifying the allocation of clinical resources to implement exercise oncology programs and for setting realistic patient expectations.

From Evidence to Implementation: The high adherence rates reported across these studies are not incidental but are a direct result of deliberate adaptations in intensity, logistics, and supervision. This translates to a clear practical implication: the successful implementation of MCT in geriatric care requires a supportive infrastructure that prioritizes professional supervision and individualization. Therefore, the next major barrier is not proving efficacy, but overcoming policy and resource constraints to integrate these programs into standard care within long-term care facilities and specialized clinics.

In summary, MCT should be considered the foundational exercise prescription for complex geriatric patients. Future work should focus on dismantling implementation barriers and refining the targeted application of adjuvant strategies like nutritional supplementation to specific, high-risk subgroups.

### The multifaceted efficacy of resistance training: from muscle to metabolism and beyond

The broader analysis of included clinical trials provides robust scientific support for the central role of RT in promoting functionality, metabolic health, and well-being among older adults. Evidence indicates that even in contexts of frailty, chronic disease, or institutionalization, regular structured RT is safe, feasible, and effective, yielding benefits across a wide spectrum of health domains.

Foundational Benefits for Muscle and Function: RT consistently produces the most robust improvements in muscular strength and functional fitness. Studies demonstrate its efficacy even in challenging populations, such as improving isokinetic strength in obese older women (23) and enhancing walking speed and functional strength in women with sarcopenia through accessible elastic-band training (3). These findings underscore its essential role in combating sarcopenia and promoting autonomy.

Cognitive and Multimodal Integration: Concerning cognitive function, findings are heterogeneous and highlight a critical nuance. While conventional RT alone shows limited cognitive effects (13), integrating cognitive tasks during training (dual-task training) proves more effective for cognitive improvement (18). This suggests that the cognitive benefits of exercise are not automatic but are contingent upon the nature of the stimulus, emphasizing the value of motor-cognitive integration.

Metabolic and Body Composition Benefits: From a metabolic perspective, RT—even at moderate intensities—effectively improves blood pressure, functional capacity, and metabolic biomarkers (11, 15). Furthermore, it is a potent stimulus for favorable body composition changes, including reductions in fat mass and increases in lean mass (9).

Feasibility and Safety Considerations: The demonstrated efficacy of simple, accessible approaches like elastic-band training (11, 15) confirms that RT can be successfully implemented with high adherence even in resource-limited settings. Nevertheless, certain precautions are warranted. Emerging evidence raises a hypothesis that high-intensity RT may induce transient cellular alterations (24), underscoring the importance of personalized, and possibly monitored, prescriptions in vulnerable populations.

In conclusion, RT serves as a cornerstone intervention for aging adults, with proven benefits for physical function, metabolism, and body composition. Its application is highly adaptable, but optimal outcomes require personalized prescription that considers individual goals, clinical status, and the nuanced evidence regarding its effects on cognitive and cellular health.

### The central dogma of resistance training: consolidating evidence and navigating future complexities

This review consolidates RT as a non-negotiable, central dogma in geriatric health. However, moving beyond this affirmation, our synthesis crystallizes key insights about its scope, limitations, and the sophisticated framework required for its optimal application.

Reaffirming Universality and Accessibility: The consistent efficacy of RT across a spectrum of modalities—from machines to elastic bands—is a finding of profound practical importance. It demonstrates that the principle of progressive overload is universally applicable, democratizing access to its benefits regardless of setting or resources. This evidence should empower clinicians to prescribe RT with confidence in diverse environments, from high-tech gyms to resource-limited nursing homes.

Defining the Boundary of Cognitive Transfer: The repeated divergence between physical and cognitive outcomes [e.g., (13, 18)] allows us to define a critical boundary: RT does not automatically confer cognitive benefits. Cognitive improvement is not a passive byproduct but an active acquisition that likely requires explicit cognitive engagement during training. This reframes the role of RT in brain health from a direct mediator to a potent platform for delivering concurrent cognitive stimulation.

The Primacy of the Exercise Stimulus Over Passive Supplements: The consistent failure of isolated micronutrient supplementation (e.g., vitamin D, omega-3) to augment the effects of RT (20) delivers a clear message. In the absence of deficiency, the active contractile stimulus is the primary and most potent driver of adaptation. This firmly places the focus—and resources—on the quality and supervision of the exercise program itself, rather than on ancillary, often ineffective, supplements.

Confronting and Contextualizing Molecular Trade-offs: The observations of potential genomic instability (24) should not be alarmist but should instead catalyze a more nuanced understanding of exercise physiology in aging. These findings hypothesize a potential molecular trade-off between the potent functional anabolism of high-intensity RT and cellular stress in very frail individuals. This underscores that “one-size-fits-all” high-intensity prescriptions are obsolete, mandating a future where exercise prescription is not only personalized to function but also informed by biomarker feedback.

In conclusion, the era of simply recommending “strength training” is over. The frontier now lies in implementing precision exercise prescription: a sophisticated integration of modality, intensity, cognitive demand, and nutritional support, all carefully calibrated to an individual’s clinical, functional, and potentially even biological profile, to maximize benefit and mitigate any nascent risks.

### Aerobic training: a foundation for endurance and systemic health, with nuanced applications

While RT is crucial for strength, this review underscores that aerobic training constitutes the foundational pillar for enhancing cardiorespiratory endurance and functional capacity in older adults. A critical synthesis, however, reveals that its role is both complementary and subject to important specificities.

The Primacy of Aerobic Exercise for Functional Mobility: The most consistent benefit of aerobic exercise lies in its unparalleled ability to improve walking capacity (e.g., 6MWT) and functional endurance. This is not merely a performance metric; it is the physiological basis for performing extended activities of daily living independently. The efficacy of even low-intensity, seated aerobic protocols highlights its unique applicability to the most frail individuals for whom high-impact or high-load activities are not feasible.

The Synergy with RT: A Non-Competitive Partnership: It is critical to frame aerobic and RT not as competitors but as synergistic partners. While aerobic exercise optimally improves central cardiorespiratory function and endurance, RT addresses peripheral musculoskeletal strength. The multicomponent interventions showing the greatest overall functional gains [e.g., (5)] validate that the combination of these two modalities produces a more comprehensive physiological adaptation than either alone.

Aerobic Exercise and Cognition: Clarifying the Pathway: The inconsistent cognitive outcomes associated with aerobic training suggest its effects are not automatic. The improvements in specific domains like executive function are hypothesized to be mediated by enhanced cerebrovascular flow and BDNF upregulation. However, this pathway appears most robust when the aerobic stimulus is coupled with an inherent cognitive challenge, such as navigating an environment or following complex routines, moving beyond simple, repetitive motions.

The Critical Role of Feasibility and Adherence: The success of home-based and low-cost aerobic interventions (e.g., using DVDs) is a testament to the critical importance of feasibility. For a behavior as crucial as sustained aerobic activity, an easily accessible, moderate-intensity program that fosters long-term adherence is often superior to an intensive, supervised program that is ultimately abandoned.

In conclusion, aerobic training is not a secondary option but a core component of geriatric exercise prescription. Its primary mandate is to sustain the cardiorespiratory engine that powers functional independence. The future of its application lies in intelligently integrating it with RT and cognitive activities to create synergistic, feasible, and personalized multimodal interventions.

In summary, despite the heterogeneity of the included literature, several key findings consistently emerge: (1) Structured exercise, particularly resistance and MCT, is a safe and effective means to improve functional fitness in older adults. (2) The most significant functional gains are often observed in frail or clinically compromised populations. (3) High adherence, facilitated by supervision and program individualization, is a critical mediator of success. (4) While physical benefits are robust, cognitive benefits are less consistent and may require specifically designed interventions that integrate cognitive challenges.

### Limitations

First, the protocol for this systematic review was not prospectively registered, which may introduce concerns regarding potential reporting bias and transparency. Second, our search was limited to three major databases (MEDLINE, PMC, and PubMed Central Canada). While this focused approach captured a substantial body of literature, it may have missed some relevant studies indexed in other databases such as Embase or CINAHL. Despite the methodological rigor of this systematic review—particularly the exclusive inclusion of RCTs and the use of validated instruments to assess functionality—several important limitations should be acknowledged. First, most included studies feature relatively small and heterogeneous samples, which may compromise the generalizability of results. Moreover, there is substantial variability in exercise protocols (duration, intensity, type, and frequency), hindering direct comparisons and the establishment of an optimal dose–response relationship. Finally, we were unable to adequately explore the potential moderating effect of individual characteristics such as sex or baseline health status on the outcomes, as the majority of included studies did not report results stratified by these factors.

## Conclusion

Structured and supervised exercise interventions, particularly those emphasizing resistance and multiple components, effectively improve functional fitness in adults aged 65 years and older. These programs enhance muscle strength, mobility, balance and, in some cases, cognitive function, contributing to greater independence and quality of life. Exercise prescriptions should be individualized, progressive and adapted to the clinical and functional status of each participant. Policymakers and healthcare providers should prioritize access to tailored exercise programs as an essential element of healthy aging strategies. Future research should focus on elucidating the optimal combination of exercise modalities, intensities and durations to maximize functional and cognitive outcomes while minimizing potential risks.

## References

[1] Rikli RE Jones CJ. Development and validation of criterion-referenced clinically relevant fitness standards for maintaining physical independence in later years. The Gerontologist. (2013) 53:255–67. doi: 10.1093/geront/gns071, 22613940

[2] World Health Organization. Decade of healthy ageing: Baseline report. Geneva: World Health Organization (2020).

[3] Seo MW Lee CW Jung HC. Effects of elastic band resistance exercise on body composition and physical function in elderly women with sarcopenia. J Exerc Rehabil. (2021) 17:55–60. doi: 10.12965/jer.2040698.349

[4] Simonsson USH Rorsman IA Axelsson J. Physical activity interventions to improve functional outcomes in aging populations: systematic review and meta-analysis. J Phys Act Health. (2023) 20:302–15. doi: 10.1123/jpah.2022-0555

[5] López-López S Abuín-Porras V Berlanga LA. Functional mobility and physical fitness are improved through a multicomponent training program in institutionalized older adults: a randomized controlled trial. Exp Gerontol. (2024) 187:112249. doi: 10.1016/j.exger.2024.112249 PMC10828358 37493861

[6] Cadore EL Rodríguez-Mañas L Sinclair A. Effects of different exercise interventions on risk of falls, gait ability, and balance in physically frail older adults: a systematic review. Rejuvenation Res. (2013) 16:105–14. doi: 10.1089/rej.2013.1397 23327448 PMC3634155

[7] Eggenberger P Theill N Holenstein S. Multicomponent physical exercise with simultaneous cognitive training to enhance dual-task walking of older adults: a secondary analysis of a 6-month randomized controlled trial with 1-year follow-up. Clin Interv Aging. (2015) 10:1711–32. doi: 10.2147/CIA.S90804 26604719 PMC4631411

[8] Stanghelle B Bentzen H Giangregorio L. Effects of resistance training in women with osteoporosis and vertebral fracture: a randomized controlled trial. Osteoporos Int. (2020) 31:1045–54. doi: 10.1007/s00198-019-05270-1

[9] Marcos-Pardo PJ Martinez-Rodriguez A Gil-Arias A. Effects of functional training on functional movement and physical fitness in older adults: a randomized controlled trial. J Aging Phys Act. (2019) 27:478–87. doi: 10.1123/japa.2018-0152

[10] Markofski MM Esser KA Panton LB. Physical activity and sarcopenia in older adults. Aging Clin Exp Res. (2019) 31:835–45. doi: 10.1007/s40520-018-1027-8

[11] Stojanović E Ristić V McMaster DT. Effects of elastic band resistance training on functional performance in older adults: a systematic review and meta-analysis. Sports Med. (2021) 51:631–54. doi: 10.1007/s40279-020-01392-8

[12] Franzke B Neubauer O Cameron-Smith D. Aging, resistance training, and DNA integrity: a review of the evidence. Front Physiol. (2020) 11:630. doi: 10.3389/fphys.2020.00630 32714198 PMC7340006

[13] Silva AF Clemente FM Roriz MS Azevedo JA Jovanovic O Adamovic M . The effect of aerobic or strength training in elderly with cognitive decline: the Fit4Alz project. Aging Clin Exp Res. (2025) 24:172–86. doi: 10.52082/jssm.2025.172, 40046223 PMC11877294

[14] Fragala MS Cadore EL Dorgo S Izquierdo M Kraemer WJ Peterson MD . Resistance training for older adults: position statement from the National Strength and conditioning association. J Strength Cond Res. (2019) 33:2019–52. doi: 10.1519/JSC.0000000000003230, 31343601

[15] Choi M Lee M Lee MJ. Effects of elastic band exercise on physical fitness and health-related quality of life in older adults. J Phys Ther Sci. (2020) 32:409–15. doi: 10.1589/jpts.32.409

[16] Lange E Kucharski D Svedlund S. Effects of aerobic and resistance exercise in older adults with rheumatoid arthritis: a randomized controlled trial. Arthritis Care Res. (2019) 71:61–70. doi: 10.1002/acr.23785 PMC6590333 29696812

[17] Lamb SE Sheehan B Atherton N. Dementia and physical activity (DAPA) trial of moderate to high intensity exercise training for people with dementia: randomized controlled trial. BMJ. (2018) 361:k1675. doi: 10.1136/bmj.k1675 29769247 PMC5953238

[18] Baek J-E Hyeon S-J Kim M Cho H-Y Hahm S-C. Effects of dual-task resistance exercise on cognition, mood, depression, functional fitness, and activities of daily living in older adults with cognitive impairment: a single-blinded, randomized controlled trial. BMC Geriatr. (2024) 24:369. doi: 10.1186/s12877-024-04942-1 38658827 PMC11044356

[19] Mueller S Winzer EB Duvinage A. Effect of high-intensity interval training, moderate continuous training, or guideline-based physical activity advice on VO_2_peak in patients with heart failure with preserved ejection fraction: a randomized clinical trial. JAMA. (2021) 325:542–51. doi: 10.1001/jama.2021.0130 33560320 PMC7873782

[20] Bischoff-Ferrari HA Vellas B Rizzoli R Kressig RW da Silva JAP Blauth M . Effect of vitamin D supplementation, Omega-3 fatty acid supplementation, or a strength-training exercise program on clinical outcomes in older adults the DO-HEALTH randomized clinical trial. J. Am. Med. Assoc. (2020) 324:1855–68. doi: 10.1001/jama.2020.16909, 33170239 PMC7656284

[21] Cho SY Roh HT. Taekwondo enhances cognitive function as a result of increased neurotrophic growth factors in elderly women. Int J Environ Res Public Health. (2019) 16:1735. doi: 10.3390/ijerph16091735, 30889827 PMC6466246

[22] Naito T Mitsunaga S Miura S. Feasibility of early multimodal interventions for elderly patients with advanced pancreatic and non-small-cell lung cancer: a prospective pilot study. Support Care Cancer. (2019) 27:577–86. doi: 10.1007/s00520-018-4353-z PMC6438328 30334618

[23] Kim SW Lee MH Lee W. Effects of 24 weeks of resistance exercise on muscle strength and physical performance in elderly women with obesity. Geriatr Gerontol Int. (2022) 22:544–50. doi: 10.1111/ggi.14395

[24] Draxler A Franzke B Strasser EM. High-intensity resistance training induces DNA damage and oxidative stress markers in elderly women: implications for training prescription. Redox Biol. (2022) 57:102490. doi: 10.1016/j.redox.2022.102490 36182809 PMC9526222

[25] Aschauer R Unterberger S Zöhrer PA Draxler A Franzke B Strasser EM . Effects of vitamin D3 supplementation and resistance training on 25-Hydroxyvitamin D status and functional performance of older adults: a randomized placebo-controlled trial. Nutrients. (2021) 14:86. doi: 10.3390/nu14010086, 35010961 PMC8746949

[26] Marcos-Pardo PJ Orquin-Castrillón FJ Gea-García GM Menayo-Antúnez R González-Gálvez N de Souza Vale RG . Effects of a moderate-to-high intensity resistance circuit training on fat mass, functional capacity, muscular strength, and quality of life in elderly: a randomized controlled trial. Sci Rep. (2019) 9:7830. doi: 10.1038/s41598-019-44329-6, 31127163 PMC6534570

